# Effect of a Phytogenic Feed Additive in Preventing Calves' Diarrhea

**DOI:** 10.3389/fvets.2022.873194

**Published:** 2022-05-11

**Authors:** Luca Turini, Alberto Mantino, Beatrice Tozzi, Francesca Bonelli, Alina Silvi, Marcello Mele, Micaela Sgorbini, Valentina Meucci, Sara Minieri

**Affiliations:** ^1^Dipartimento di Scienze Agrarie, Alimentari, Agro-ambientali, University of Pisa, Pisa, Italy; ^2^Centro di Ricerche Agro-Ambientali “E. Avanzi”, University of Pisa, Pisa, Italy; ^3^Istituto di Scienze della Vita, Sant'Anna School of Advanced Studies, Pisa, Italy; ^4^Dipartimento di Scienze Veterinarie, University of Pisa, Pisa, Italy

**Keywords:** calves, diarrhea, phenols, tannins, phytotherapy

## Abstract

The aims of the present study were to evaluate the preventive and the therapeutic effect of Stodi^®^ as phytogenic feed additive rich in phenolic substances on the calf diarrhea, during the first 24 days of life. A total of 40 calves were included and randomly divided into Group C (control group) and Group T (treated group) with placebo or treatment administration started from the third day of life (T0). Calves belonged to group C received 2 L of warm water, while the calves assigned to group T received 2L of warm water plus 30 g of Stodi^®^. Solutions administration was maintained until day 21 (T21) that was the end of the experimental period. Calves were weighed at T0 and T21 to assess the average daily gain (ADG). Physical examination and fecal score evaluation were performed daily. The duration of a diarrheic episode, the age of the first diarrhea outbreak (TDE) and the frequency of diarrheic episodes were recorded. Complete blood count, methemoglobin and liver enzymes were evaluated at T0 and at T21 in all the calves by spectrophotometer and clinical chemistry analysis, respectively. Data were analyzed using a mixed model. A Chi-square and a Mann-Whitney test were also performed. No difference was found for ADG between the groups. The difference of mean age at TDE was not statistically significant between C and T group. The number of calves with diarrhea in the C group tended to be higher than that of T group (*p* = 0.13). Calves in group C spent more days with clinical sign of diarrhea compared to group T (*p* = 0.016). Complete blood count, methemoglobin and liver enzymes were within the reference ranges. The feed additive Stodi^®^ seemed to be effective in shortening neonatal diarrhea episodes in calves thanks to the administration of 30 g per day of product. The fixed dosage of Stodi^®^ used in our study did not show a preventive effect to reduce the incidence of calf diarrhea.

## Introduction

Calf diarrhea results from a complex interaction between the environment, animal factors and infectious agents, such as Rotavirus, Coronavirus, *Cryptosporidium parvum* and *Escherichia coli*, representing one of the most common causes of mortality in dairy calves (1). Calf diarrhea and its sequela may deeply impact the profitability of a dairy farm by decreasing calves' growth rate and lifetime productivity and increasing treatment costs (2). Calves are usually affected by diarrheic episode between 0- and 4-week-old, with a peak within 2 weeks of age (3). As an alternative to antibiotics, phytotherapy has grown more popular in the treatment of a variety of diseases in both human and veterinary medicine. The wide use of antibiotics in the food chain can reduce the efficacy of therapeutically used antibiotics and increase the risks for human infections caused by antibiotic-resistant bacteria (4). Thus, an alternative method to improve animal health and the relative performance is to provide additives that minimize the risk of diseases. Polyphenols are known for their astringent and anti-inflammatory properties on the gastrointestinal tract (5). Several studies evaluated polyphenols substances, pure or as mixture for gastrointestinal diseases in piglets, horses and cattle (6–8). In neonatal calves infected with *Cryptosporidium parvum*, Oliveira et al. (9) used pomegranate-residue supplements to examine a reduction in the fecal oocyst count, as well as the intensity and duration of diarrhea. More recent studies evaluated the reduction of the diarrheic episode following the therapeutic (8) and the preventive (10) administration of chestnut tannins in a population of sick calves. Stodi^®^ (Garzanti Specialties, Milano, Italy) is a blend of several Indian medicinal plants, rich in phenolic substances, traditionally known and being used for gut health and contains fruit rinds of Punica granatum, aerial part of Andrographis paniculate, bark of Acacia Nilotic, fruits of Terminalia bellirica, and bark of Holarrhena antidysentery. According to Marimuthu et al. (11), adding Stodi^®^ to the diet of broilers was efficient in altering the cecal microbial community in a way that was beneficial to gut health and performance, as evidenced by an increase in the quantity of beneficial microflora. Due to the increasing interest in studying alternative products for antimicrobial treatment in livestock, the present study aimed to evaluate both the preventive and therapeutic effect of Stodi^®^ as phytogenic feed additive rich in phenolic substances on the calf diarrhea, during the first 21 days of life.

## Materials and Methods

The Institutional Animal Care and Use Committee (OPBA, Pisa, Prot. n. 33479/2016) approved this prospective blinded cohort study, which was conducted at the University of Pisa's dairy farm (Centro di Ricerche Agro-Ambientali “E. Avanzi”), where approximately 120 animals were kept in free-stall conditions. All the procedures were in compliance with the 2010/63/EU directive regarding the protection of animals in scientific experiments.

### Experimental Design

During the study period, a total of 40 calves aged between 3 and 24 days were enrolled. Calves were Italian Friesian and cross breed calves Italian Friesian x Limousine. At birth, calves were randomly assigned to Group C (control group) (*n* = 20) or to Group T (treated group) (*n* = 20). All the calves were maintained adopting the same management condition. Briefly, within 30 min after calving, the calf was removed from the dam, weighed with a scale (ID 3000, Tru-Test Limited, USA), and identified. The calves were subsequently placed in a single straw-bedded pen (2.5 x 2 m) where they could interact with one another, in accordance with European Legislation (2008/119/CE). Within 1.5 h after calving, colostrum was collected from the dam and evaluated using an optical Brix refractometer (Atago brix N1, Japan). Colostrum higher than 21% Brix scale was classified as high quality, according to the literature (12). A total of 3 L of the dam's colostrum was administered with a nipple bucket between 30 min and 2 h after birth. Further 3 L were administered within the next 8 h (13). An operator observed if the calves ate all the colostrum administered. From the third day of life, a total of 6 L of pool milk was administered twice a day for all the calves involved in the experimental study.

Twenty-four hours after colostrum feeding, serum total protein (TP) was evaluated with a refractometer designed specifically for this purpose (AR200; Reichert Analytical Instruments, Reichert Inc., Depew, NY), in order to assess the passive transfer of immunity resulted from the colostrum ingestion; failure of passive transfer was defined as a serum TP score below 5.5 g/dL (14).

Starting on the third day of life (T0), all the calves in group C received 2 L of warm water q24h, while the calves in group T received 2L of warm water plus 30 g of Stodi^®^ q24h; solutions administration was maintained until day 21 (T21) that was the end of the experimental period. In order to assess the average daily gain (ADG), calves were weighed at T0 and T21.

Both solutions were administered by two expert operators at the same time (10.00 in the morning) using a graduated calf bottle which was equipped with a flexible rubber nipple specifically designed for calf feeding. The bottle was first filled with warm water, and gave to calves of Group C, while the soluble powder was added for calves of Group T. After every administration, the bottle was opened and checked in order to ensure the entire intake.

Physical examination included evaluation from a distance plus a hands-on examination, focusing on physical appearance, body weight, body condition, head, mouth, eyes, ears, neck and back, thorax, abdomen, umbilicus, musculoskeletal system, perianal region, body temperature, feces, urine, and external genitalia (15). Milk intake was also recorded throughout the whole study period. The diagnosis of diarrhea was based on fecal score (FS) evaluation which represents an assessment of fecal fluidity. Fecal score evaluation was made every day, individually, ready before the administration of the solutions along with the physical examination already described (16). Score 0 = normal consistency; score 1 = semi-formed or pasty; 2 = loose but enough consistency to stay on bedding; 3 = watery feces that leak through bedding material, according to literature (17). Diarrhea was defined as a FS ≥ 2 (16). Contextually, the dehydration status was also assessed (18).

The FS was documented everyday by the same professional operator by examining fresh feces in the pen starting on the first day of diarrhea. A blinded procedure was applied for the FS evaluation. The operator in charge for FS assessing was unaware of the calf's ID and calf's group. The period (in days) from the first diarrhea outbreak (fecal score 2) and the normalization of the FS (fecal score = 0 or 1) was defined as the duration of a diarrheic episode (DDE). The age expressed in days of the first diarrhea outbreak (TDE) and the frequency of diarrheic episodes defined as the number of episodes per calf throughout the whole study period were also recorded. At the beginning of the diarrhea, a fresh fecal sample was collected from each calf by manual restraint in two different sterile tubes: a rapid ELISA test was performed on one aliquot right away (test strips for detection of Rotavirus, Coronavirus, *E. coli* F5 and *C. parvum* in bovine feces, Biox Diagnostics, Belgium), while the second aliquot was stored in a refrigerated bag and evaluated within 1 h for gastrointestinal parasites (1). Starting from the onset of the diarrhea until the resolution of the episode, calves in both groups received 1 tablet of Effydral^®^ (Italy Zoetis Ltd.) (sodium chloride 2.34g, potassium chloride 1.12g, sodium bicarbonate 6.72g, citric acid anhydrous 3.84g, lactose monohydrate 32.44g, glycine 2.25g) as an oral rehydration solution in 2 L of water q24h (1).

Grass hay was ad libitum available for all the calves after the first week of life. All of the feeding procedures were carried out by a professional operator utilizing a nipple bucket. Even when there were cases of diarrhea, there were still no feeding restrictions or adjustments in feeding regime.

### Inclusion Criteria

The inclusion criteria were calves born during the period of the study (July 2019–July 2020) that showed a successful transfer of passive immunity 24 h after birth. Calves were excluded from the study if the passive transfer of immunity was inadequate (serum TP score below 5.5 g/dL), if they showed diarrhea before the third day of life, or if they were affected by a disease other than diarrhea during their first 1 month of age.

### Laboratory Analysis

Analysis of the STODI^®^ mixture was performed in order to obtain the content of total phenols, simple phenols, total tannins and condensed tannins (Table 1). The analysis was done according to following procedures:

**Table 1:** Characterization of phenolic compounds contained in the STODI^®^ mixture.

**Compounds**	**Mean**	**SD**
Total phenols (mg tannic acid/g dry weight)	57.86	6.99
Simple phenols (mg tannic acid/g dry weight)	6.6	0.82
Total tannins (mg tannic acid/g dry weight)	51.26	6.35
Condensed tannins (mg catechin/g dry weight)	3.88	0.72

#### Phenolic Extraction

About 0.05 g of the STODI^®^ mixture (*n* = 5) was homogenized with 2 mL of 80% (v/v) methanol solution by sonication for 30 min, keeping the temperature within the range 0 to 4°C. After centrifugation (6,000 g for 10 min at 4°C), supernatants were collected and passed through PTFE (Polytetrafluoroethylene; 0.20 μm pore size; Sarstedt, Verona, Italy). Extracts were stored at −80°C before analysis (19).

#### Measurement of Total Phenols and Tannins

The contents of total phenols and total tannins were determined using the Folin–Ciocalteau reagent, as described by Katoch (20).

Briefly, 10 μL of phenolic extract was added to 490 μL of ultrapure H2O and 250 μL of Folin–Ciocalteau reagent and then 1.25 mL of the sodium carbonate solution. The absorbance at 725 nm was recorded after 40 min. The total phenols were calculated as tannic acid equivalent by using the following calibration curve:


$$\begin{array}{l} Y\;=\;0.0537x\;+\;0.0027\;R2\;=\;0.99\text{.} \end{array}$$

About 100 mg of PVPP was added to 1.0 mL of distilled water and then 1.0 mL of the phenolic extract in order to precipitate tannins. The solution was vortexed and kept at 4°C for 15 min, then the mixture was centrifuged (3,000 g for 10 min) and the supernatant was collected. The supernatant was analyzed again with the Folin–Ciocalteu method by using 100 μL of extract in order to obtain total simple phenols.

The total tannins were calculated as follows:


$$\begin{array}{l} \mathrm{Totaltannins}=\mathrm{TPC}-\mathrm{TSP} \end{array}$$

were TPC: Total Phenol content, TSP: Total Simple Phenols.

#### Measurement of Condensed Tannins

Condensed tannins were estimated by using vanillin-HCl method as reported by Salar and Pulewar (21). 1.5 mL of vanillin (4 % w/v) reagent was added into 100 μL of previous phenolic extracts followed by addition of 750 μL of concentrated hydrochloric acid. The resulting mixture was vortexed and then allowed to stand at room temperature for 20 min. At 500 nm, the absorbance against a blank was measured. Catechin was used to make the standard curve (0.05–1 mg/mL).


$$\begin{array}{l} Y\;=\;0.0393x\;+\;0.0004\;R2\;=\;0.99 \end{array}$$

#### Blood Analysis

Complete blood count (CBC), blood methemoglobin, serum albumin (ALB), gamma-glutamyl transferase (GGT) and aspartate aminotransferase (AST) were evaluated at T0 and at T21 in all the calves. Blood samples were drawn from the jugular vein in both EDTA Vacutainer tubes (2 mL × 2 tubes) and Vacutainer tubes without EDTA (2.5 mL) (Becton Dickinson and Co., Franklin Lakes, NJ) and immediately refrigerated until the analysis. Blood sample for CBC was analyzed by a cell counter (ProCyte Dx, IDEXX) within 5 min of collection. Serum ALB, GGT and AST were evaluated within 3 hours after collection using the clinical chemistry and immunoturbidimetric analyzer Liasys© (AMS, Alliance, Italy), while methemoglobin was evaluated according to Bonelli et al. (10).

### Statistical Analysis of Results

The SAS software (SAS 8.0, SAS Institute, 1999) was adopted for the statistical analysis. Data about calf weight, average daily gain and hematologic parameters were analyzed with a Shapiro-Wilk normality test in order to assess data distribution. Data were analyzed by a mixed model including treatment, sampling time, their interaction and sex as fixed factors and animal as random factor. Due to the not normally distribution of the data, results concerning the frequency of diarrheic episode and fecal score were expressed as median value (minimum and maximum value) and a Mann-Whitney test was performed to evaluate difference between the two groups. A Chi-square test was performed to compare the total amount of days spent into diarrhea during the whole study period between the two groups. Statistical significance was assigned to values having a *P*-value of <0.05.

## Results

All the calves born during the experimental period were enrolled (*n* = 40) because they met the inclusion criteria; thus, 20 calves were randomly included in the Group C and 20 calves were included in the Group T. Group C was composed by 16/20 (80%) female calves (13 Italian Friesian calves and 3 cross breed calves), and 4/20 (20%) male calves (all cross breed calves), while Group T was composed by 17/20 female calves (85%) (14 Italian Friesian calves and 3 cross breed calves) and 3/20 (15%) male calves (all cross breed calves). No calves refused the polyphenols solution. No pharmacological treatments or fluid therapy were needed because calves never showed alterations upon physical examination or milk intake, and the hydration status was within the physiologic parameters.

After the application of the rapid ELISA test for the identification of the etiological agent of infection, 11% of calves from both group C and group T were found to be positive for *Cryptosporidium parvum*, whereas 22% of calves belonging to group T were positive for Rotavirus. Eighty-eight percent of calves belonging to group C and 66% belonging to group T were negative to the rapid ELISA test. Intestinal parasites were not found using fecal flotation.

The average weight at the end of the study period (W-21) was 52.35 ± 1.08 kg and 56.78 ± 1.27 kg for groups C and T, respectively. The ADG was 0.67 ± 0.03 kg/day, and 0.71 ± 0.07 kg/day, for groups C and T, respectively. There were no significant differences between the two groups. No significant differences have been found between sex for any variable considered.

The difference of mean age at TDE was not statistically significant between C and T group (9.8 ± 1.5 days vs 7.4 ± 1.5 days for C and T group, respectively).

**Figure 1:**
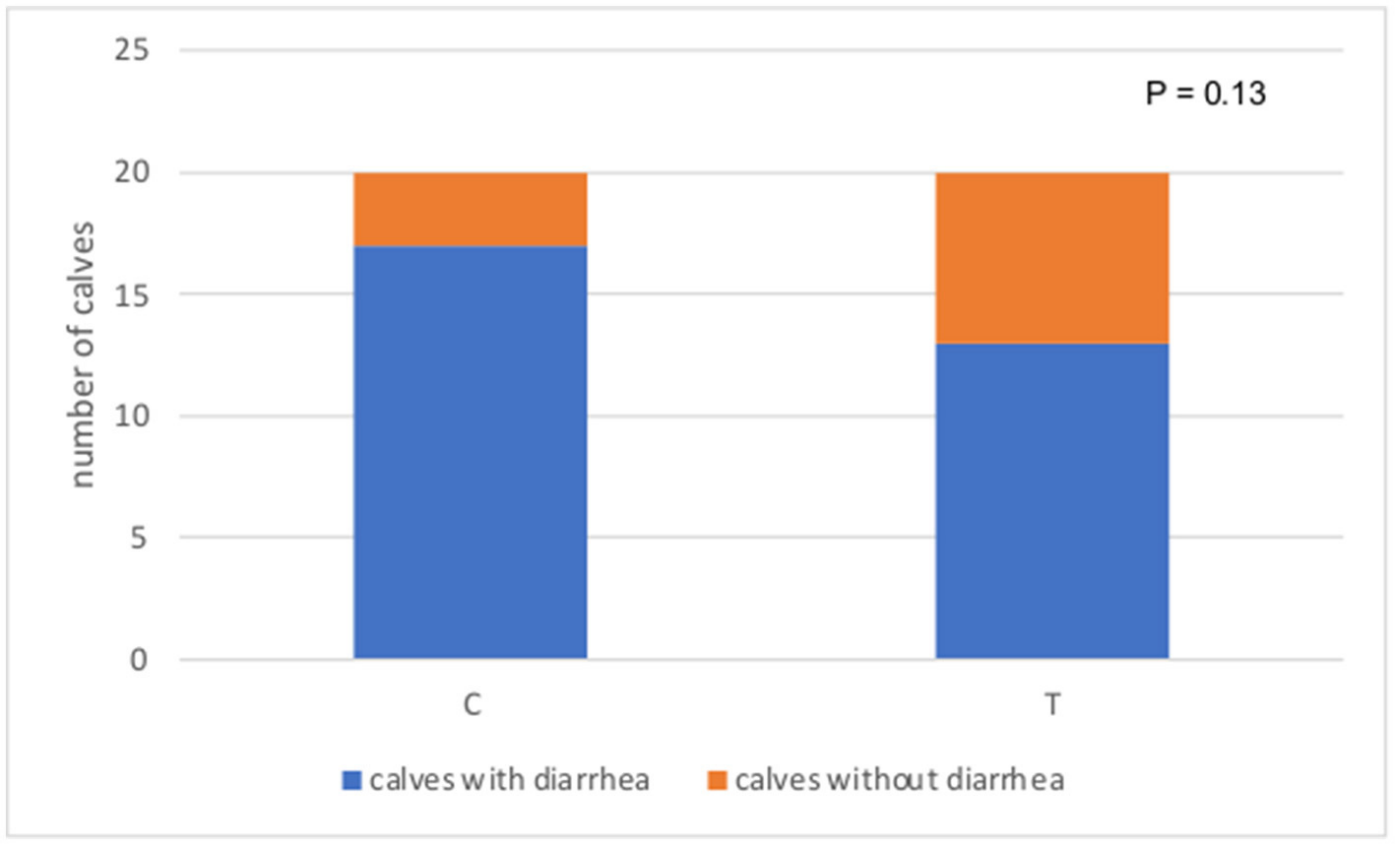
Number of calves with diarrhea in the control (C) and treatment group (T).

**Figure 2:**
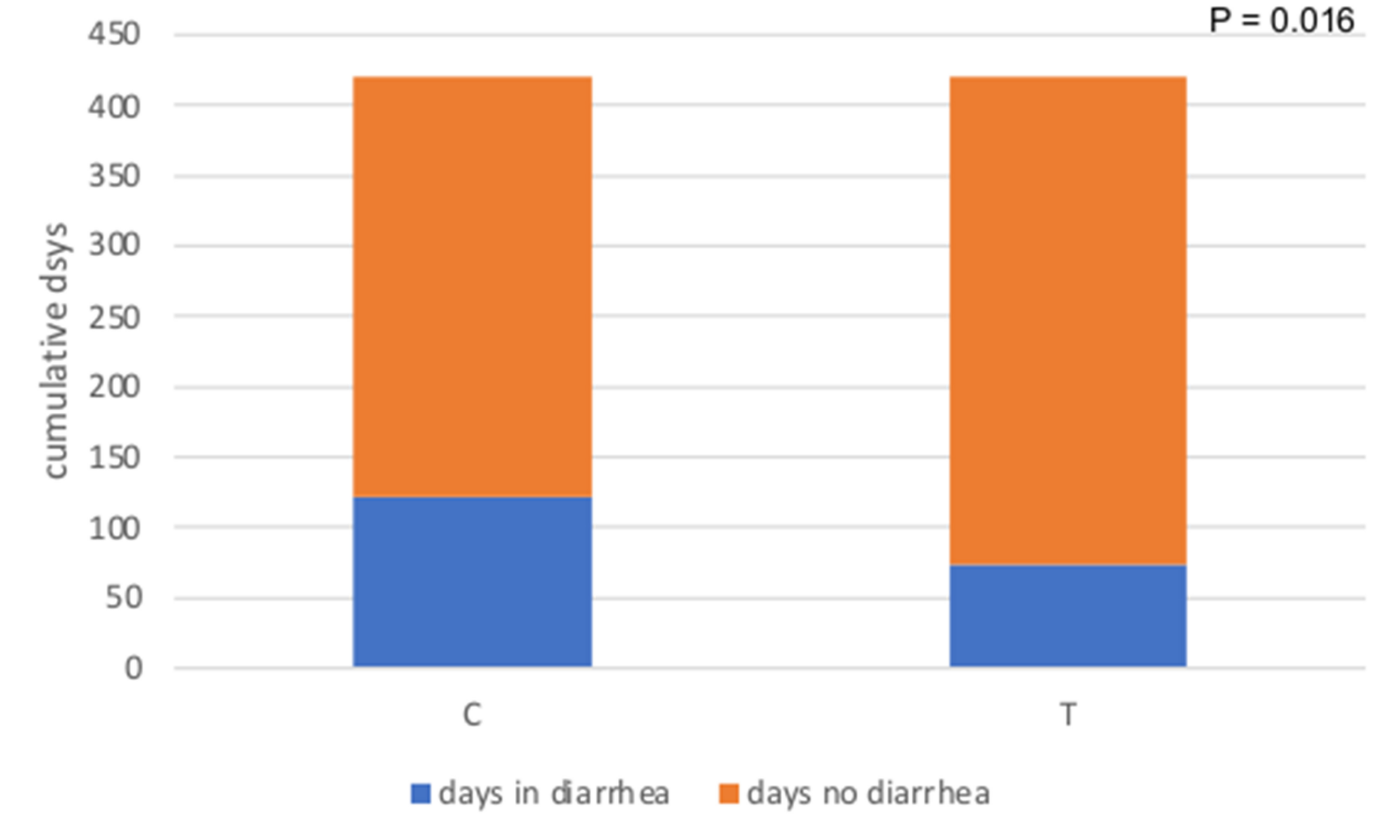
Cumulative number of days spent in diarrhea in the control (C) and treatment group (T).

The number of calves with diarrhea in the C group tended to be higher than that of T group (17 vs. 13 for C and T group, respectively, *P* = 0.13) (Figure 1). When considering the total number of days in the experiment (21 days × 20 calves per group), calves in group C spent 28.8% of the time (121 days) with clinical signs of diarrhea, whereas calves in group T had clinical signs of diarrhea for 17.6% of the time (nearly 74 days), and the difference between the two groups was significant (*P* = 0.016) (Figure 2). The median fecal score was 2 (*m* – 2 and *M* – 3) for both groups. Data on CBC and ALB, AST and GGT values at time 0 and time 21 for both experimental groups are reported in Tables 2, 3, respectively. All the values were within the reference ranges (22). The values of red blood cells (RBC), hematocrit (HCT), hemoglobin (HGB), mean cell volume (MCV) and reticulocytes were statistically higher at T0, compared to T21 in both groups. In both group C and T, the values of mean corpuscular hemoglobin concentration (MCHC) and red blood cells distribution width (RDW) were statistically lower at T0 compared to T21. Results about neutrophils and eosinophils were statistically higher at T0 compared to T21, while lymphocytes were statistically lower at T0 than at T21. For both groups values of ALB were statistically higher at T21 compared to T0, while GGT concentrations were lower. Data on methemoglobin were within the reference ranges (23) and no significant differences were found between groups.

**Table 2:** Results of complete blood count (CBC) expressed as LSmean for group C (*n* = 20 calves) and group T (n 20 calves) at 0 and 21 days of life.

	**Group C**		**Group T**		**SEM**	***P*** **-value**		
	**T0**	**T21**	**T0**	**T21**		**Treatment**	**Time**	**Interaction**
RBC (M/μL)	7.07	6.17	7.43	6.75	0.385	0.311	0.011	0.711
HCT (%)	27.89	21.66	30.57	21.99	1.769	0.487	0.001	0.368
HGB (g/dL)	8.77	7.47	9.49	7.77	0.497	0.385	0.001	0.600
MCV (fL)	38.59	34.46	40.95	32.38	0.867	0.877	0.001	0.411
MCH (pg)	11.86	12.17	12.76	11.58	0.353	0.641	0.254	0.154
MCHC (g/dL)	30.46	35.59	31.21	36.80	1.207	0.461	0.001	0.831
RDW %	31.22	38.13	30.70	38.76	1.054	0.959	0.001	0.585
RETIC (K/μL)	5.92	0.63	6.29	0.38	1.256	0.963	0.001	0.810
WBC (K/μL)	7.43	7.79	7.18	8.37	0.508	0.778	0.082	0.351
NEU (K/μL)	4.83	3.05	5.06	3.64	0.417	0.379	0.001	0.637
LYM (K/μL)	2.15	3.94	1.74	4.34	0.246	0.980	0.001	0.052
MONO (K/μL)	0.05	0.34	0.02	0.27	0.038	0.202	0.001	0.628
EOS (K/μL)	0.05	0.02	0.06	0.03	0.009	0.461	0.002	0.748
BASO (K/μL)	0.35	0.44	0.31	0.41	0.081	0.053	0.329	0.424

*C, control group; T, polyphenols-treated group; SEM, standard error of mean; RBC, red blood cells; HCT, hematocrit; HGB, hemoglobin; MCV, mean corpuscular volume; MCHC, mean corpuscular hemoglobin concentration; RDW, red cell distribution width; RETIC, reticulocytes; WBC, white blood cells; NEU, neutrophils; LYM, lymphocytes; MONO, monocytes; EOS, eosinophils; BASO, basophils*.

**Table 3:** Results of albumin (ALB), aspartate aminotransferase (AST) and gamma-glutamyl transferase (GGT) concentration expressed as LSmean for group C (*n* = 20 calves) and group T (*n* = 20 calves) at 0 and 21 days of life.

	**Group C**		**Group T**		**SEM**	***P*** **-value**		
	**T0**	**T21**	**T0**	**T21**		**Treatment**	**Time**	**Interaction**
ALB (U/L)	2.54	2.95	2.53	2.99	0.061	0.727	<0.001	0.678
AST (U/L)	48.53	43.95	47.82	52.05	5.561	0.556	0.975	0.,441
GGT (U/L)	526.77	152.39	631.84	139.78	87.022	0.604	<0.001	0.509

*C, control group; T, polyphenols-treated group; SEM, standard error of mean*.

## Discussion

Since 2006, the European Union has restricted the use of antimicrobials and minerals boosting the research for feed additives in farm animals, aimed to maintain or improve animal health and the related performance (4). Natural compounds such as plant secondary metabolites, showed a wide range of beneficial effects on animal's health. Polyphenols, a group of plant secondary metabolites, have been postulated for anti-bloat qualities, improved nitrogen utilization, reduced methane generation and the anti-helminthic effects (24).

Calf diarrhea may deeply impact calves' growth rate and lifetime productivity, increasing costs for the farmer. Moreover, the sequela of calf diarrhea may lead to an increased use of drugs, including antimicrobials (2). Due to the increasing interest for natural compounds as an alternative to antimicrobials in livestock production, the aim of the present study was to evaluate the effect of a mixture of Indian medicinal plants rich in phenolic substances on calf diarrhea. The phenolic composition of the plant mixture is largely based on tannins that accounted for more than 88% of total phenols (Table 1). Condensed tannins were also present, accounting for nearly 4 mg per gram of dry weight. On the basis of the total phenol concentration found in the feed additive (nearly 58 mg/g of dry weight), the amount of total phenols daily administered to the calves was nearly 1.7 g of tannic acid equivalent. In literature, the daily dose of polyphenols administered to calves varied from 1.7 g/d to 7.5 g/d (9, 10), depending to the nature of polyphenol and the purity of the plant extract adopted. Since the mixture adopted in the present study was not previously tested on calves, we assumed as safe the lower level reported in the literature.

The feed additive appeared to be pleasant for all the calves enrolled in the study and was easy to administer for the operators. This represents an interesting result, consistent with previous papers about calves and pure tannins administration (8, 10), because one of the most important characteristics for an oral solution is the palatability and the ease in feeding it to livestock.

Rotavirus and Cryptosporidium parvum were the pathogens discovered by the rapid ELISA test in our calves. According to literature, they represent, along with Coronavirus and enterotoxigenic *E. coli*, the most common infectious causes of diarrhea in neonatal calves (1). However, we also recorded a high percentage of animals negative to the rapid ELISA test. The assay used in the present study for the etiological diagnosis of calf's diarrhea showed a very high sensibility for *C. parvum* (25). However, this assay is unable to detect Cryptosporidium species other than *C. parvum* and despite neonatal diarrhea is generally caused by *C. parvum* in calves, it is not possible to exclude that other Cryptosporidium species were involved in the etiology of some diarrhea events (26). This peculiarity of the assay might have influenced results about the prevalence of infectious agents isolated.

The average weight at the end of the study period (W-21) and the ADG were in line with literature (27). The lack of significant differences in ADG between the two groups suggested that the polyphenols powder does not affect the animals' growth and was in line with a study based on similar amounts of pomegranate tannins administration to calves (9). However, other studies supplementing calves with higher amounts of polyphenols did not report significant effects on ADG in the first 60 days of life in calves (8, 10).

Considering the whole observation period, the percentage of days spent in diarrhea by the calves of group T was significantly lower than for group C (17.6% vs. 28.8%). These results were in line with findings reported in other studies on calves (8, 10, 28) where the shortening of the diarrheic episode was found in polyphenols-treated animals. Previous studies reported that polyphenolic compounds or polyphenol-rich plant extracts suppress experimentally induced inflammation processes, especially in the gut (29). Polyphenols' anti-inflammatory effects are mediated by a variety of biological pathways, the majority of which are tied to NF-jB inhibition (30). The ability of activation of several antioxidant enzymes by polyphenols could also be an explanation of the results found in the present study (31). Spending a lower number of days in diarrhea can reduce medical complications such as dehydration, hematogenous spread of pathogens, lactic acidosis, and decreased feed intake. These conditions normally required an intensive care approach, aggressive fluid therapy and use of antimicrobials (32). Using a polyphenol-rich additive since the third day of life might reduce the calf diarrhea sequela decreasing costs for farmers and leading to a better future performance of animals.

No significant differences were found concerning the onset of diarrhea and the average fecal score during the diarrheic episode, however the number of calves experiencing diarrhea tended to be higher in the control group (Figure 1). Literature is controversial: some studies showed a therapeutic effect of polyphenols if they were administered in calves affected by neonatal diarrhea (8, 28, 33). Treated animals presented an earlier improvement of the fecal consistency score (33) and the onset of diarrhea was later than in the control group (10). Overall, effects on prevention of neonatal calves' diarrhea are not reported (9, 28). Differences about the tannin efficacy of calf diarrhea may be related to the different biochemical composition of the product used. Moreover, we administered the polyphenols powder at the same dose throughout the whole study period despite the calf's growth rate. This might have influenced some results since a dose-dependent effect in improving the diarrhea episode was found in a recent study (28).

Concerning blood parameters, all the values were within the reference ranges (22, 34) and no differences were found between the two experimental groups, while some differences were recorded between sampling times. The values of RBC, HCT, HGB, MCV and reticulocytes decreased significantly from T0 to T21, in agreement with previous studies in newborn calves (34, 35). This may be related to hemodilution, physiological destruction of erythrocytes by the spleen and/or decreased erythropoietin production secondary to increased blood oxygenation by lungs after birth, as already reported in foals (36). In both group C and T, the values of MCHC and RDW increased from T0 to T21. Blood concentrations of RDW were generally higher in calves compared to adult cows (37); literature reported that MCHC values of calves remain constant until the 5th week of age and rise significantly thereafter (37). In the present study, the increasing of MCHC seemed to happen earlier compared to literature. This may be due to factors able to influence the CBC such as age, gender, breed, environment, and management practices (38). Neutrophils and eosinophils values decreased statistically from T0 to T21, while lymphocytes increased. Within the first hours after birth, a decrease in the blood concentrations of neutrophils and eosinophils was found, while the number of lymphocytes increased. These changes observed in the calves' leukograms within the first week of life have been mainly attributed to the stress of labor and birth and to a natural adaptation to the extra-uterine life (38). Calves of both groups presented higher concentrations of ALB and lower levels of GGT from T0 to T21, while no differences were found between groups and for AST and methemoglobin between time. Due to the lack of differences between the two groups, the differences between time were probably related to age and not to treatment. Albumin levels partially reflect hepatic synthesis (35). They are predominantly synthesized in the liver; thus, their amount depends on the maturity and functional ability of the liver (39). Newborns showed an immaturity of both the hepatic anabolic pathways and kidney glomerular function which led to lower albumin concentrations around birth (40). With time, albumin production increased due to hepatic maturation, albumin loss decreased due to kidney maturation; these mechanisms lead to increasing serum albumin levels in older calves, as seen in our data in both groups. The circulating GGT levels can be physiologically increased by colostrum feeding in ruminants (22).

Despite the timing discrepancies, the serum AST, ALB, and GGT levels, as well as blood methemoglobin levels, were all within normal limits, corroborating prior findings in neonatal calves (10).

In conclusion, the dietary addition of a feed additive rich in phenolic substances seemed to be effective in shortening neonatal diarrhea episodes in calves thanks to the administration of 30 g per day of product without any adverse metabolic effects. At a constant dosage, no preventive effect was detected for the etiologic agents found in our study population. Further research could investigate the preventive aspect by giving the calves a customized dose based on their daily weight gain.

## Data Availability Statement

The raw data supporting the conclusions of this article will be made available by the authors, without undue reservation.

## Ethics Statement

The animal study was reviewed and approved by the Institutional Animal Care and Use Committee (OPBA, Pisa, Prot. no. 33479/2016).

## Author Contributions

LT: conceptualization, methodology, and writing—original draft preparation. AM and BT: resources and data curation. FB: methodology, data curation, and writing—review and editing. AS: validation and investigation. MM: supervision, software, project administration, and writing—review and editing. MS: writing—review and editing. VM: formal analysis and data curation. SM: supervision, funding acquisition, and writing—review and editing. All authors contributed to the article and approved the submitted version.

## Funding

This research was supported by Garzanti Specialties, Milano, Italy (Grant Number: 0002869/2018).

## Conflict of Interest

The authors declare that the research was conducted in the absence of any commercial or financial relationships that could be construed as a potential conflict of interest.

## Publisher's Note

All claims expressed in this article are solely those of the authors and do not necessarily represent those of their affiliated organizations, or those of the publisher, the editors and the reviewers. Any product that may be evaluated in this article, or claim that may be made by its manufacturer, is not guaranteed or endorsed by the publisher.
